# Latent trajectories of adaptive behaviour in infants at high and low familial risk for autism spectrum disorder

**DOI:** 10.1186/s13229-019-0264-6

**Published:** 2019-03-15

**Authors:** Giorgia Bussu, Emily J. H. Jones, Tony Charman, Mark H. Johnson, Jan K. Buitelaar, BASIS Team, Anna Blasi, Simon Baron-Cohen, Rachael Bedford, Patrick Bolton, Susie Chandler, Celeste Cheung, Kim Davies, Janice Fernandes, Isobel Gammer, Holly Garwood, Jeanne Giraud, Anna Gui, Kristelle Hudry, Michelle Lieu, Evelyne Mercure, Sarah Lloyd-Fox, Helen Maris, Louise O’Hara, Andrew Pickles, Helena Ribeiro, Erica Salomone, Leslie Tucker, Agnes Volein

**Affiliations:** 1 Department of Cognitive Neuroscience, Donders Institute for Brain, Cognition and Behaviour, Radboud University Medical Center, Kapittelweg 29, 6525 EN Nijmegen, The Netherlands; 2 Centre for Brain and Cognitive Development, Birkbeck College, University of London, London, UK; 3 Department of Psychology, Institute of Psychiatry, Psychology and Neuroscience, King’s College London, London, UK; 4 South London and Maudsley NHS Foundation Trust (SLaM), London, UK; 5 Department of Psychology, University of Cambridge, Cambridge, UK; 6 Karakter Child and Adolescent Psychiatry University Centre, Nijmegen, The Netherlands

**Keywords:** Autism, Trajectories, Adaptive behaviour, Infant siblings, Subgroups

## Abstract

**Background:**

Autism spectrum disorder (ASD) is characterised by persisting difficulties in everyday functioning. Adaptive behaviour is heterogeneous across individuals with ASD, and it is not clear to what extent early development of adaptive behaviour relates to ASD outcome in toddlerhood. This study aims to identify subgroups of infants based on early development of adaptive skills and investigate their association with later ASD outcome.

**Methods:**

Adaptive behaviour was assessed on infants at high (*n* = 166) and low (*n* = 74) familial risk for ASD between 8 and 36 months using the Vineland Adaptive Behavior Scales (VABS-II). The four domains of VABS-II were modelled in parallel using growth mixture modelling to identify distinct classes of infants based on adaptive behaviour. Then, we associated class membership with clinical outcome and ASD symptoms at 36 months and longitudinal measures of cognitive development.

**Results:**

We observed three classes characterised by decreasing trajectories below age-appropriate norms (8.3%), stable trajectories around age-appropriate norms (73.8%), and increasing trajectories reaching average scores by age 2 (17.9%). Infants with declining adaptive behaviour had a higher risk (odds ratio (OR) = 4.40; confidence interval (CI) 1.90; 12.98) for ASD and higher parent-reported symptoms in the social, communication, and repetitive behaviour domains at 36 months. Furthermore, there was a discrepancy between adaptive and cognitive functioning as the class with improving adaptive skills showed stable cognitive development around average scores.

**Conclusions:**

Findings confirm the heterogeneity of trajectories of adaptive functioning in infancy, with a higher risk for ASD in toddlerhood linked to a plateau in the development of adaptive functioning after the first year of life.

**Electronic supplementary material:**

The online version of this article (10.1186/s13229-019-0264-6) contains supplementary material, which is available to authorized users.

## Background

Autism spectrum disorder (ASD) is a set of heterogeneous developmental disorders characterised by difficulties in the social-communication domain, restricted and repetitive patterns of behaviours and interests, and sensory anomalies [1]. A diagnosis of ASD according to the DSM-5 criteria further requires that symptoms cause clinically significant impairments in everyday functioning. The resulting long-term outcome is mixed, but many children diagnosed with ASD will have a suboptimal quality of life in adulthood [2–4], with persisting impairments in everyday functioning [5]. Adaptive behaviour reflects the ability of an individual to function independently in everyday situations and reflects how an individual responds to environmental demands translating capacities into everyday competencies. Evidence suggests that adaptive behaviour in ASD is heterogeneous across individuals and even within the same individual over time [6]. A useful approach to explore this heterogeneity is growth mixture modelling (GMM). GMM is a data-driven approach which allows one to identify distinct mixtures of trajectories within population defining classes of individual growth curves [7]. Previous studies have used GMM to parse samples based on the heterogeneity in the development of adaptive behaviour, mainly focusing on pre-school and school-aged children with ASD [6, 8–11]. These studies analysed approximately 100 children with ASD to derive classes of developmental trajectories on the Adaptive Behaviour Composite (ABC) score [8, 9] and the Daily Living score [10, 11] of the Vineland Adaptive Behavior Scale (VABS-II [12]). Only one study [6] used a larger sample to identify classes in ABC scores (*n* = 421). Results mainly showed one class with low functioning and decreasing scores, another class with moderate functioning and stable scores over time, and one final class with higher functioning and substantial increase of scores over time. The partial replication of classes across these studies suggests that developmental trajectories of adaptive skills may actually serve to define subgroups in the ASD phenotype.

The work reviewed above focused on children from approximately 3 years of age who already had a diagnosis of ASD. Thus, it could not capture the different developmental trajectories preceding an ASD diagnosis. The investigation of early development of adaptive skills is critical to predict later functional outcome and then enable early targeted intervention. Early access to personalised interventions can, in turn, be crucial to improve later functioning in different environments of everyday life through learning of adaptive skills [13]. This stresses the importance of investigating not only the presence and development of ASD symptoms, which can even be masked by learned strategies to cope with environmental demands [1], but also the different developmental pathways of adaptive behaviour that infants can follow early in life.

However, adaptive behaviour has been rarely used so far as an outcome measure in prospective research. A recent study used mixed models to examine developmental trajectories of cognitive and adaptive functioning between 7 months and 7 years of age, measured respectively by the Mullen Scales of Early Learning (MSEL) and the VABS-II, in infants at familial high-risk (HR) and low-risk (LR) for ASD [14]. Results showed that HR siblings who met the criteria for ASD at age 7 had increasing difficulties in adaptive behaviour over time compared to LR controls, while HR siblings without later ASD outcome did not differ in adaptive behaviour from LR siblings. These findings extend previous work on the same dataset [15] and independent work from Estes and colleagues [16] on trajectories of adaptive behaviour between 8 and 36 months. These studies showed decreased adaptive functioning by 12 months in high-risk siblings developing ASD compared to non-ASD siblings and low-risk controls. There is a partial overlap in data between the study from Salomone et al. [14] and the present study; however, analytic methods and research aims were different. In fact, although these findings improve our understanding of the different developmental profiles of adaptive functioning in infancy and toddlerhood, analyses were based on clinical outcome groups and might not have captured the variation in phenotypes within clinical categories. Only one recent study has investigated latent trajectories of adaptive functioning in a high-risk cohort [17]. This study analysed 566 infants between 12 and 36 months to derive classes of developmental trajectories on the ABC score. Results showed one class with average scores at 12 months and a declining trajectory, one class with a slightly declining trajectory, and one class with higher scores and a stable trajectory.

The current study aimed to identify distinct classes of infants based on the early development of adaptive functioning in HR siblings and LR controls. We used a prospective analysis approach to discover structure in data, independently from clinical categories. Specifically, we used parallel process GMM to simultaneously examine communication, daily living, motor, and social domains of adaptive behaviour, and observe strengths and impairments in the different areas of everyday functioning. Then, we characterised the identified classes in terms of ASD outcome and symptoms at 36 months and concurrent trajectories of cognitive development. Previous studies have shown a discrepancy between adaptive functioning, cognitive abilities, and ASD symptoms in older children with ASD [6, 18–20]. These findings suggest that neither normative cognitive development nor low levels of ASD symptoms are protective factors against poor adaptive functioning. Our post hoc association of class membership with cognitive development and symptom severity allowed us to investigate the relationship between these three areas of functioning in early development. Overall, the exploratory investigation of inter-individual heterogeneity with such an unsupervised approach provides better insight into the variety of paths leading to different functional outcomes within ASD and typical development.

## Methods

### Participants and procedure

Data were collected from 247 infants from the British Autism Study of Infant Siblings [21], across two phases of the study based on time of recruitment. Infants were considered at high (*n* = 170) and low (*n* = 77) familial risk for ASD based on having or not an older biological sibling with ASD. Fifty-four high-risk and 50 low-risk infants participated to phase 1 [22], while an independent cohort of 116 HR and 27 LR participated to phase 2. LR controls were full-term infants recruited from a volunteer database at the Birkbeck Centre for Brain and Cognitive Development. At the time of enrolment, none of the infants had been diagnosed with any developmental condition. Infants were followed longitudinally on four visits: 8 months (mean = 8.1, standard deviation (SD) = 1.2), 14 months (mean = 14.5, SD = 1.3), 24 months (mean = 25.0, SD = 1.8), and 36 months (mean = 38.8, SD = 3.0). To allow testing for quadratic growth, the final sample included infants with data available from at least 3 visits, leading to a final sample of 240 infants (74 LR and 166 HR). Study researchers were aware of infants’ risk status but were blind to clinical outcome.

### Measures

#### Adaptive functioning

The Vineland Adaptive Behavior Scale (VABS-II [12]) is a semi-structured parent-report questionnaire (at 8 and 14 months) or parent interview (at 24 and 36 months) assessing infant’s adaptive behaviour in everyday settings. The items address personal and social functioning in four different domains (standard scores; mean = 100, SD = 15): communication (Comm), daily living skills (DL), socialisation (Soc), and motor abilities (Mot). Standard scores from the 4 domains between 8 and 36 months were included in our main analysis to identify homogeneous classes of infants.

#### Developmental skills

Verbal and non-verbal cognitive development was measured at each visit by the Mullen Scales of Early Learning (MSEL [23]), a standardised developmental measure used to assess cognitive functioning between birth and 68 months. Scores are obtained in the following five domains: gross motor (GM), visual reception (VR), fine motor (FM), receptive (RL), and expressive language skills (EL). The Mullen Scale provides normative scores for each scale (*T*-scores mean = 50, SD = 10) and a single composite score representing general intelligence (early learning composite (ELC) standard score mean = 100, SD = 15). ELC scores between 8 and 36 months were included in our analyses to characterise the developmental level of the identified classes.

#### Early ASD symptoms

The Autism Diagnostic Observation Schedule (ADOS [24]), a standardised diagnostic instrument; the Autism Diagnostic Interview-Revised (ADI-R [25]), a structured parent interview; and the Social Communication Questionnaire (SCQ [26]), a screening tool for ASD, were administered at 36 months to assess autism symptoms. Of note, the ADI-R was not administered to LR infants from phase 1 (*n* = 47) and missing values were imputed through expectation maximisation on SPSS [27].

To evaluate the end level of symptom severity of the identified classes, we included in our analysis the ADOS Calibrated Severity Score (CSS) obtained from the raw total scores (CSS-Tot), and Social Affect (CSS-SA) and Restricted and Repetitive Behavior (CSS-RRB) domains; the ADI-R domain scores for the Social (ADI-Soc), Communication (ADI-Comm), and Repetitive Behaviors and Interests domains (ADI-RBI); and the SCQ total score (SCQ-Tot).

### Clinical outcome

The LR group was based on having an older full sibling with typical development. LR infants received no formal clinical diagnoses, but none of them had a community clinical ASD diagnosis at 36 months. In particular, no ADI-R was administered to LR in phase 1, who did not receive an outcome evaluation. In phase 2, LR infants were administered the ADOS and ADI-R and received an outcome evaluation at 36 months, but none of them raised any concern for ASD or atypical development. HR siblings received a clinical outcome evaluation at 36 months and were subsequently grouped into siblings with ASD (HR-ASD), with atypical (non-ASD) development (HR-Atypical), and with typical development (HR-Typical).

Expert clinical researchers reviewed all available information at 24 months and 36 months and assigned clinical consensus best estimate diagnosis of ASD (HR-ASD) according to ICD-10 [28] or DSM-5 criteria depending on the recruitment phase [1]. Diagnoses were reviewed for differences in categorisation and considered to be similar. Among high-risk infants who did not meet criteria for ASD, a subgroup of siblings was classified as “atypical” (HR-Atypical) based on ADOS and/or ADI-R above ASD threshold, and/or MSEL more than 1.5 standard deviations below average on visual reception and/or receptive language and/or expressive language and/or early learning composite (*n* = 15) scores. Finally, siblings who did not meet the criteria for ASD or atypical development were classified as HR-Typical.

### Data analysis: an overview

We used a three-step approach to identify latent classes of adaptive behaviour and profile them through associations with external variables. First, the four domains of the Vineland were modelled in parallel through growth mixture modelling to identify latent class trajectories of adaptive behaviour on 4 time-points between 8 and 36 months. Second, infants were assigned to latent classes based on posterior probabilities of class membership. Third, the identified classes were characterised in terms of clinical outcome and symptom severity at 36 months and longitudinal cognitive development.

### Identification of latent class trajectories

We chose growth mixture modelling to identify distinct mixtures of trajectories within population. As opposed to other methods such as latent class growth curve modelling [29], which assumes a homogeneous pattern of behaviour within class, growth mixture modelling [7] allowed us to capture the complexity of adaptive behaviour in developmental variation across individuals.

We investigated the pattern of missing data for the four domains of adaptive behaviour by testing its association with gender and clinical outcome at 36 months. Differences in gender were not significant, while the proportion of missing data at 24 months was significantly dependent on clinical outcome at 36 months (*χ*^2^ [3] = 8.23, *p* = 0.04), with HR-Atypical having most missing data. However, differences in outcome were not significant at other time-points, providing reasonable evidence for a pattern of data missing at random. Thus, individuals with missing data were included in the analysis, allowing us to use all available information. In fact, individual trajectories of adaptive behaviour were modelled on data available at an individual level.

Real age was included as a fixed effect while random effects on intercept and slope were modelled on an individual level. Multiple models were tested based on the polynomial degree of the growth curve, the variance/covariance matrix, and the number of classes. Models were run with 1 to 6 classes, and each class number was run separately 50 times to control for local maxima. The best model was determined in terms of data fitting and parsimony based on having lower values of Bayesian information criterion (BIC), Akaike information criterion (AIC), negative log-likelihood, and higher average class posterior probability [30]. Analyses were performed using the *multlcmm* function from the *lcmm* package in R [31].

The classes derived from parallel process growth mixture modelling were subsequently compared on adaptive behaviour over time through hierarchical mixture modelling [32]. A quadratic mixed model was tested with VABS-II domain scores as outcome variables and real age and class membership as fixed factors, while gender was included as a covariate and random effects on intercept and slope were modelled on an individual level. We investigated the main effects of *class*, *age*, *age*^2^ and their interaction effects using Wald tests with Satterthwaite approximation for degrees of freedom. Post hoc Tukey’s tests for multiple comparisons were performed for class comparisons and simple main effects analysis. Analyses were implemented using the *lme4* software package on R [33].

### Characterisation of latent classes

Classes in adaptive behaviour, as derived from parallel process growth mixture modelling, were further characterised by examining the association of class membership with independent outcome variables. First, we examined the association with ASD symptom severity at 36 months, as measured by the CSS-Tot, CSS-SA, CSS-RRB, ADI-Comm, ADI-Soc, ADI-RBI, and SCQ-Tot scores, through an analysis of variance. For significant differences, classes were compared through post hoc Tukey’s tests for multiple comparisons.

Then, we examined the association of class membership with trajectories of cognitive development, as measured by the MSEL ELC score between 8 and 36 months, through hierarchical mixture modelling [32]. Models were built using the *lme4* software package on R [33], with MSEL ELC scores as outcome variables, real age and class membership as fixed factors, and gender as a covariate, while random effects on intercept and slope were modelled on an individual level. We compared linear and quadratic models on age to select the best fit based on chi-squared tests on the log-likelihood values.

## Results

Among HR siblings at 36 months, 34/166 (20.5%) siblings were categorised as HR-ASD, 44/166 (26.5%) as HR-Atypical, and 87/166 (52.4%) as HR-Typical. Among HR-Atypical, 32/87 and 6/87 siblings, respectively, had ADOS and ADI-R scores above ASD threshold, 9/87 siblings had MSEL more than 1.5 standard deviations below average on visual reception, 14/87 on receptive language, 9/87 on expressive language, and 15/87 on early learning composite scores. Finally, 1/166 infant sibling did not receive a clinical outcome evaluation but was included in our trajectory analysis having complete data on adaptive behaviour. Descriptive statistics for the entire sample and the classes identified are shown in Table 1, while descriptive statistics by risk group are reported in Additional file 1: Table S1.

**Table 1:** Descriptive statistics

	Tot		C1 decreasing adaptive behaviour		C2 average/stable adaptive behaviour		C3 recovering adaptive behaviour	
	Mean	SD	Mean	SD	Mean	SD	Mean	SD
Age								
8 m	8.1	1.2	7.9	1.5	7.9	1.1	8.7	1.3
14 m	14.4	1.3	14.1	1.4	14.3	1.2	15.1	1.4
24 m	25.0	1.8	25.7	2.7	25.0	1.8	25.0	1.5
36 m	38.8	2.9	40.2	3.6	38.6	3.0	38.9	2.2
VABS								
Comm 8 m	96.1	15.9	108.2	14.7	99.2	12.6	77.0	14.2
Comm 14 m	96.7	13.3	91.1	15.9	98.0	13.1	93.4	11.8
Comm 24 m	103.7	13.0	88.7	20.1	104.7	11.3	106.6	11.2
Comm 36 m	101.1	14.1	86.8	19.8	102.2	12.9	102.9	12.6
DL 8 m	100.1	13.7	106.7	18.4	101.3	12.3	91.9	13.8
DL 14 m	95.2	13.1	90.6	17.8	96.5	13.0	91.7	10.0
DL 24 m	105.7	12.9	94.4	20.0	106.7	11.9	106.7	10.1
DL 36 m	103.2	13.0	87.9	20.9	104.1	11.5	106.2	9.3
Mot 8 m	89.8	16.3	98.4	24.2	91.8	14.5	76.9	12.7
Mot 14 m	100.3	12.8	99.1	14.9	101.9	12.5	94.3	11.4
Mot 24 m	100.1	10.9	90.2	12.0	101.2	10.4	100.4	10.1
Mot 36 m	93.8	12.4	84.6	14.7	94.7	11.9	94.1	11.7
Soc 8 m	100.1	12.8	109.6	19.7	101.4	10.6	90.0	11.7
Soc 14 m	97.7	11.7	97.5	13.8	98.7	11.8	93.6	9.7
Soc 24 m	101.0	11.6	89.1	18.0	101.5	10.6	104.1	8.7
Soc 36 m	97.8	12.9	85.6	17.9	98.3	12.1	101.2	10.5
MSEL								
ELC 8 m	104.2	15.0	106.2	18.2	104.9	14.7	100.4	14.6
ELC 14 m	98.5	16.0	86.2	19.3	100.6	15.7	95.6	12.6
ELC 24 m	104.7	19.9	81.9	25.8	106.8	18.0	105.7	19.1
ELC 36 m	107.7	22.7	89.6	26.7	108.6	22.7	111.9	17.4
ADOS at 36 m^1^								
CSS-Tot	2.95	2.40	3.84	3.00	2.94	2.43	2.60	1.90
CSS-SA	3.40	2.51	4.00	2.89	3.43	2.55	3.02	2.14
CSS-RRB	4.29	2.61	5.42	2.73	4.14	2.58	4.42	2.62
ADI-R at 36 m^2^								
ADI-Comm^3^	3.25	4.17	7.00	5.75	2.96	3.85	2.67	3.81
ADI-Soc^3^	3.08	4.31	7.55	7.76	2.66	3.68	2.67	3.35
ADI-RBI^4^	1.15	1.98	2.40	2.58	1.12	1.93	0.70	1.63
SCQ at 36 m^2^								
SCQ-Tot^3^	5.32	6.11	11.1	8.83	5.02	5.64	3.91	5.03
Clinical outcome at 36 m^5^	*n* (%)		*n* (%)		*n*^6^ (%)		*n* (%)	
LR	74 (31)		4 (20)		56 (32)		14 (32)	
HR-Typ	87 (36)		6 (30)		63 (36)		18 (42)	
HR-Atyp	44 (18)		2 (10)		34 (19)		8 (19)	
HR-ASD	34 (14)		8 (40)		23 (13)		3 (7)	
Gender								
Female	122 (51)		7 (35)		92 (52)		23 (53)	
Male	118 (49)		13 (65)		85 (48)		20 (47)	

This table shows the descriptive statistics for the entire sample and for the different trajectory classes of adaptive behaviour

*Tot* entire sample, *C1–C3* classes in trajectories of adaptive behaviour^7^, *VABS* Vineland Adaptive Behavior Scales, *Comm* communication score, *DL* daily living score, *Mot* motor score, *Soc* socialisation score, *MSEL* Mullen Scales of Early Learning, *ELC* early learning composite score, *ADOS* Autism Diagnostic Observation Schedule, *CSS* Calibrated Severity Score, *ADI-R* Autism Diagnostic Interview-Revised, *ADI-Comm* Communication domain score (ADI-R), *ADI-Soc* Social domain score (ADI-R), *ADI-RBI* Restricted Behaviors and Interests domain score (ADI-R), *SCQ* Social Communication Questionnaire, *SCQ-Tot* total score (SCQ), *LR* low-risk controls, *HR* high-risk siblings, *HR-Typ* typically developing siblings, *HR-Atyp* atypically developing siblings (no ASD), *HR-ASD* siblings with ASD

^1^Data were available for a subsample of *n* = 235 infants

^2^Data were available for a subsample of *n* = 239 infants

^3^Significant difference per class with *p* < 0.001

^4^Significant difference per class with *p* < 0.05

^5^Clinical outcome vs class membership: *χ*^2^ [6] = 13.39, *p* = 0.037

^6^One infant in this class did not receive a clinical outcome evaluation at 36 months

^7^C1, decreasing adaptive behaviour; C2, average/stable adaptive behaviour; C3, recovering adaptive behaviour

**Fig. 1:**
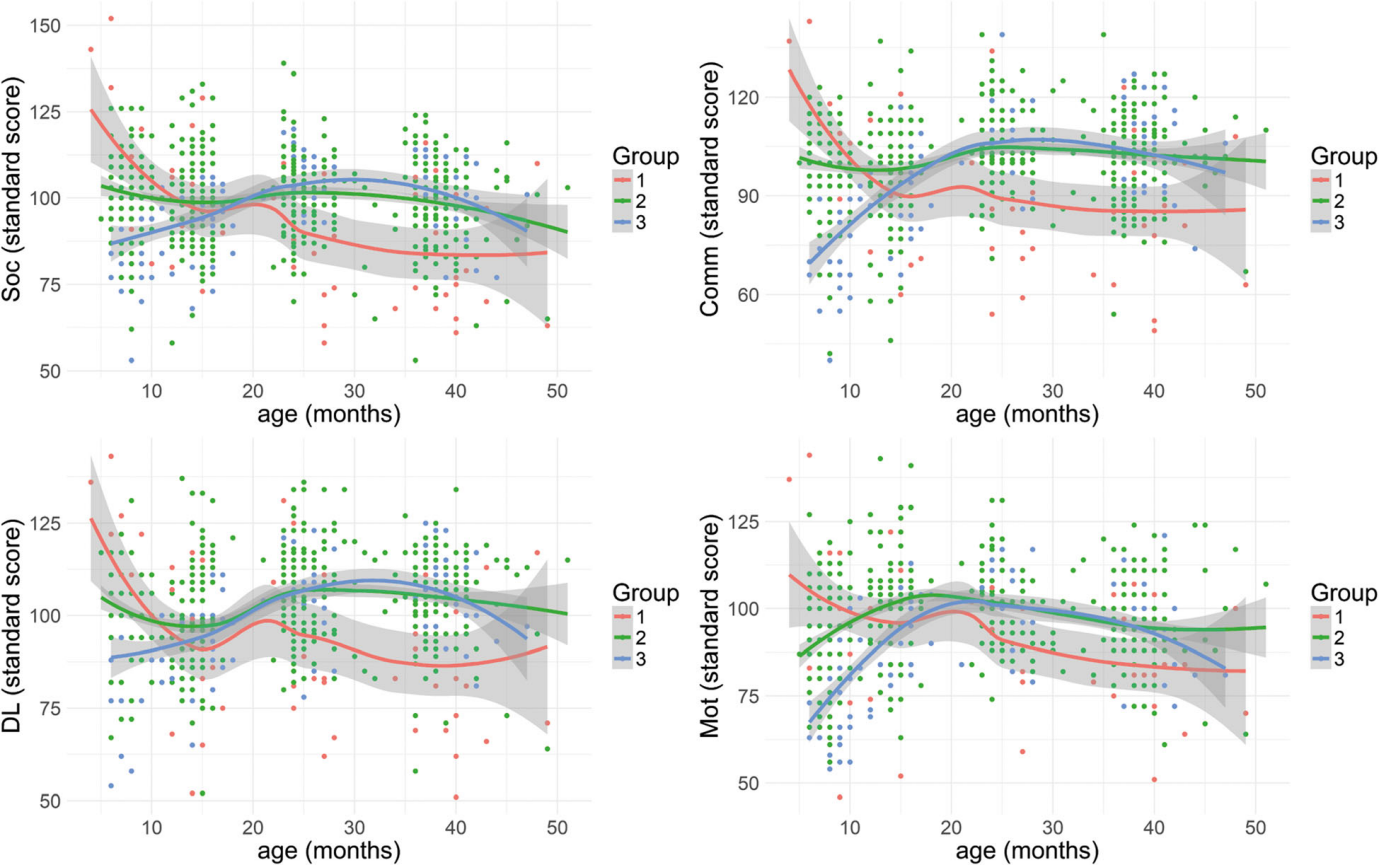
This figure illustrates the three classes identified in longitudinal adaptive behaviour. Points show individual scores, while classes were computed through the *loess* function in R for visualisation purposes. Class 1 shows a decreasing trajectory in all domains; class 2 shows a stable trajectory at an average level of adaptive behaviour; class 3 shows recovering (improving) trajectories, starting from low scores in all domains and reaching an average level from around 20 months onwards.

**Fig. 2:**
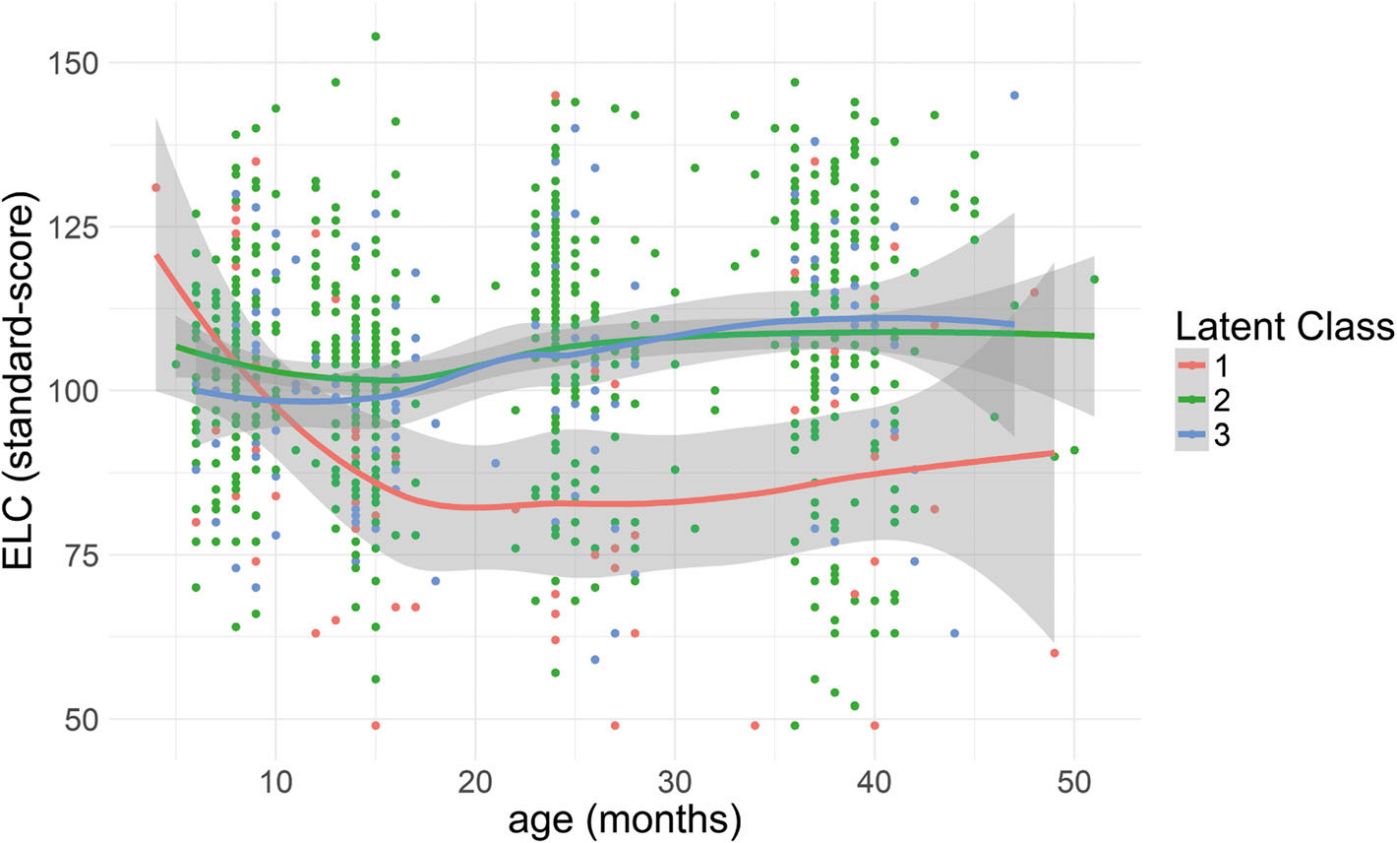
Developmental trajectories in cognitive level

Three classes of quadratic trajectories provided the best fit to the data, with BIC = 28,523.12 and AIC = 28,397.82 and average posterior probability of 87%. Metrics of model fitting are reported in Additional file 1: Table S2. The identified trajectories of adaptive behaviour are shown in Fig. 1. Modelling the corresponding trajectories in ELC scores (Fig. 2), the quadratic model was the best fit for the data (*χ*^2^ [6] = 26.2, *p* < 0.001).

This figure illustrates the developmental trajectories of cognitive level, as measured by the ELC score from the Mullen Scales of Early Learning, for the three classes identified in longitudinal adaptive behaviour. Points show individual scores while classes were computed through the *loess* function in R for visualisation purposes. Class 1 shows a decreasing trajectory in cognitive level; classes 2 and 3 show stable trajectories at an average level of cognitive development.]

Class 1 (*n*_1_ = 20 (8.3%)) shows decreasing trajectories in all domains of adaptive behaviour (fixed effect of age in the common longitudinal model: *β* = − 0.27, standard error (SE) = 0.08, *p* = 0.001) starting with above average age-standardised scores in communication, daily living, and socialisation skills and average scores in motor skills in the first year of life. This is the only class with unbalanced gender, being more males (65%) than females (35%). Results from linear mixed modelling show that infants in class 1 had significantly decreasing standardised scores in communication, daily living, and socialisation domains between 8 and 24 months (*p* < 0.001), and in socialisation between 24 and 36 months (*p* = 0.04), while motor scores decreased significantly only between 14 and 24 months (*p* = 0.02). Infants in this class also show a similar trajectory in cognitive development over time, with decreasing ELC scores before age 2 (*p* < 0.001), starting from above average scores in the first year of life.

Class 2 (*n*_2_ = 177 (73.8%)) shows stable trajectories around average scores in all domains of adaptive behaviour (fixed effect of age in the common longitudinal model: *β* = 0.11, SE = 0.03, *p* < 0.001). Gender was balanced in this class, with 48% of males and 52% of females. Results from linear mixed modelling show that infants had significantly increasing age-standardised scores between 8 and 14 months in communication (*p* = 0.004) and daily living domains (*p* = 0.04); increasing scores between 14 and 24 months in communication, daily living, and motor domains (*p* < 0.001); and decreasing motor scores between 24 and 36 months (*p* < 0.001). Infants in this class show a stable trajectory around average scores in cognitive development before age 2 and increasing ELC scores afterwards (*p* = 0.005).

Class 3 (*n*_3_ = 43 (17.9%)) shows recovering (improving) trajectories (fixed effect of age in the common longitudinal model: *β* = 0.43, SE = 0.10, *p* < 0.001) starting from low scores and reaching a stable average level in all domains by age 2. Gender was balanced in this class, with 47% of males and 53% of females. Results from linear mixed modelling show that infants had significantly increasing age-standardised scores between 8 and 24 months in all domains of adaptive behaviour (*p* < 0.001). Infants in this class also show a stable trajectory around average scores in cognitive development before age 2 with increasing ELC scores afterwards (*p* = 0.007).

Mixed models on trajectories of adaptive behaviour show that class 3 had significantly lower scores than classes 1 and 2 in all domains at 8 months (p < 0.001), while it had only significantly lower scores than class 2 in communication and motor scores at 14 months (*p* < 0.001). Class 1 had significantly lower scores than classes 2 and 3 in communication (*p* < 0.001), daily living (*p* = 0.002 and *p* = 0.006, respectively), motor (*p* = 0.004 and *p* < 0.05, respectively), and socialisation scores (*p* = 0.006 and *p* = 0.002, respectively) at 24 months. Finally, class 1 had significantly lower scores than classes 1 and 2 in communication (*p* < 0.001), daily living (*p* < 0.001), motor (*p* = 0.002 and *p* = 0.007, respectively), and socialisation scores (*p* < 0.001) at 36 months (see Fig. 1).

### Relationship between classes and cognitive development

Infants in class 1 had significantly lower scores on cognitive development, measured by the MSEL, than those in class 2 at 14 months (*p* = 0.01), in classes 2 (*p* < 0.001) and 3 (*p* = 0.002) at 24 months, and in classes 2 and 3 at 36 months (*p* < 0.001), while classes 2 and 3 identified from the longitudinal development of adaptive behaviour did not differ in cognitive development (see Fig. 2). Thus, there was a split between adaptive skills and cognitive skills for the improving class.

### Relationship between classes and clinical outcome at 36 months

Clinical outcome was mixed in all identified classes. The distribution of outcome in each class is reported in Table 1. Although HR-ASD development was not specific to any class, a *χ*^2^ test revealed a significant relationship between class membership and clinical outcome (*χ*^2^ [6] = 13.39; *p* = 0.037). In particular, there were significantly more HR-ASD siblings in class 1 compared to the other classes (odds ratio for HR-ASD in class 1 compared to class 3: OR = 4.40 (CI 1.90; 12.98), *p* < 0.001). Although classes did not differ in ADOS CSS-Tot (*p* = 0.17) nor in ADOS domain scores at 36 months (CSS-SA: *p* = 0.35, CSS-RRB: *p* = 0.12), differences were significant in ADI-Comm (*F*(2,236) = 9.56, *p* < 0.001), ADI-Soc (*F*(2,236) = 13.0, *p* < 0.001), ADI-RBI (*F*(2,236) = 5.32, *p* = 0.005), and SCQ-Tot scores at 36 months (*F*(2,236) = 11.19, *p* < 0.001). Post hoc comparisons showed higher symptom severity for infants in class 1 compared to the other classes (*p* < 0.001 for all scores except for ADI-RBI showing *p* < 0.05), while differences were not significant between class 2 and class 3 (Table 2). Thus, class membership is significantly related to ASD symptoms and clinical outcome, with higher symptom severity and a higher risk for ASD in class 1.

**Table 2:** Class differences in symptom severity at 36 months

	ANOVA			Post hoc Tukey’s tests					
				Class 2 vs. class 1		Class 3 vs. class 1		Class 3 vs. class 2	
	*F*	dof	*p*	*t*	*p*	*t*	*p*	*t*	*p*
ADOS at 36m^1^									
CSS-Tot	1.77	2/232	0.17	–	–	–	–	–	–
CSS-SA	1.04	2/232	0.35	–	–	–	–	–	–
CSS-RRB	2.15	2/232	0.12	–	–	–	–	–	–
ADI-R at 36m^2^									
ADI-Comm^3^	9.56	2/236	< 0.001^*^	− 4.25	< 0.001^*^	− 3.97	< 0.001^*^	− 0.42	0.91
ADI-Soc^3^	13.0	2/236	< 0.001^*^	− 5.04	< 0.00^*^	− 4.39	< 0.001^*^	0.01	1.00
ADI-RBI^4^	5.32	2/236	0.005^*^	− 2.79	0.015^*^	− 3.23	0.004^*^	− 1.28	0.40
SCQ at 36m^2^									
SCQ-Tot^3^	11.2	2/236	< 0.001^*^	− 4.40	< 0.001^*^	− 4.53	< 0.001^*^	0.50	0.50

This table shows the detailed statistics from the ANOVA-investigated differences between classes in symptom severity at 36 months as measured by the ADOS, ADI-R, and SCQ. When differences were significant, post hoc Tukey’s tests for multiple comparisons were performed for paired comparisons of classes. Differences were considered significant for *p* < 0.05 (marked as “*”)

*ANOVA* analysis of variance, *Class 1* decreasing adaptive behaviour, *Class 2* average/stable adaptive behaviour, *Class 3* recovering adaptive behavior, *dof* degrees of freedom, *ADOS* Autism Diagnostic Observation Schedule, *CSS* Calibrated Severity Score, *ADI-R* Autism Diagnostic Interview-Revised, *SCQ* Social Communication Questionnaire

^1^Data were available for a subsample of *n* = 235 infants

^2^Data were available for a subsample of *n* = 239 infants

^3^Significant difference per class with *p* < 0.001

^4^Significant difference per class with *p* < 0.05

## Discussion

This study explored latent trajectories of adaptive functioning in infants at high and low familial risk for ASD. We observed variability in the development of adaptive functioning before age 2 and found three latent classes: one class with scores at or above age-appropriate norms at the first visit but decreasing trajectories afterwards (class 1), one class with a relatively stable trajectory around age-appropriate norms (class 2), and one class with increasing scores from below age-appropriate norms before age 2 to stable average scores afterwards (class 3). Thus, high adaptive skills early in development were counterintuitively associated with poorer adaptive functioning in toddlerhood, while an initially delayed development appeared to be recovered by age 2. From age 2 onwards, the identified classes mainly showed one relatively stable trajectory around average scores and a below-average decreasing trajectory, consistent with previous findings on older children with ASD [6, 8, 9]. Of note, classes significantly differed in ASD symptom severity and clinical outcome. Infants in class 1 had significantly higher symptom severity at 36 months, and there were more siblings who later met the criteria for ASD than expected by a randomly distributed ASD outcome. Another important finding was a partial split between adaptive behaviour and cognitive development of the identified classes. In fact, class 3 showed a stable trajectory around average cognitive level while it was identified by improving adaptive behaviour before age 2 and a stable trajectory around average scores afterwards.

Classes did not clearly map to clinical outcome groups. In our sample, 68% of infants developing ASD showed rather stable trajectories of adaptive skills around age-appropriate norms (class 2), 23% of them showed decreasing skills (class 1), and only 9% of them showed recovering adaptive skills by age 2 (class 3). Similarly, the HR-Atypical outcome group was spread over the three classes, although the majority was in class 2. This provides further support to the high heterogeneity of ASD in its phenotypic manifestations [34–36]. Yet, it is in contrast with our previous work on group comparisons, showing significantly lower adaptive skills in HR-ASD siblings compared to HR-Typical or LR controls and overall stable-low or decreasing trajectories of adaptive behaviour in infancy [14, 15]. However, our approach here is focused on the identification of latent classes of trajectories independently from diagnostic outcome. Such approach allowed us to explore individual differences in early development and to identify different profiles within the ASD group.

Different trajectories of adaptive behaviour did not correspond to significant differences in ADOS scores, but there were significant differences in ADI-R and SCQ scores. This extends to infancy previous findings on discrepancies between adaptive behaviour and ADOS scores in pre-schoolers with ASD [6]. However, not all ASD symptoms are captured by the ADOS, and some children with “atypical” outcomes had by definition high ADOS scores (but did not meet diagnostic criteria for ASD). Rather, the relationship between adaptive behaviour and symptom severity can be captured by investigating all the instruments employed to assess symptom severity at 36 months (i.e. ADOS, ADI-R, and SCQ). The split between ADI-R/SCQ and ADOS might be due to the parent-reported nature of ADI-R and SCQ scores, the same as VABS scores used to identify classes of infants, while ADOS scores are based on clinical observations.

It is remarkable to observe initially average or above-average adaptive functioning in some infants with later ASD outcome (class 1). Nevertheless, they also showed a decreasing trajectory of scores compared to age-appropriate norms over time, which is in line with previous findings on children with ASD [6, 8]. Furthermore, the timing of the decline observed here is consistent with the emergence of overt behavioural signs of ASD as generally observed in previous studies around the second year of life [37–39]. Thus, an initial high level of adaptive functioning seems not to prevent ASD development; rather, when followed by a decline over time, it seems to be associated with a higher likelihood of ASD in toddlerhood. However, given that class 1 represents only 7% of infants in our sample, we must be cautious with interpretations. Our findings are somewhat similar to what is observed in neuroimaging studies. There, higher fractional anisotropy and volume in the development of fibre tracts [40, 41], accelerated expansion of cortical surface area, and brain volume overgrowth [42] in the first year of life were linked to later ASD outcome. One may speculate that high levels of adaptive skills early in development are linked to early alterations of brain development. In particular, hyper-expansion of cortical surface area may compromise the development of proper neural connectivity [43] and have downstream effects on behaviour, leading to the emergence of symptoms characteristic of ASD in toddlerhood. A recent framework for neurodevelopmental disorders suggests, in fact, that good synaptic compensation to overcome initial impairment at an earlier developmental stage might have more severe consequences later in development [44]. The declining trajectory of adaptive skills that we observed likely reflects little gain of skills between the first and second year of life and a failure to keep up with development. Further research should integrate more biological data on brain structure and brain function, such as EEG or MRI, to investigate what could have triggered the plateauing of skills for infants in class 1. This would also provide more insight into the biological mechanisms underlying the identified developmental trajectories. Furthermore, while classes 2 and 3 were balanced in gender, class 1 was mostly composed by males. This is consistent with the gender bias in the prevalence of ASD [45] and with previous findings in children with ASD showing higher daily living scores in females than males [46]. Overall, this suggests that sex might moderate how clinical symptoms are expressed in adaptive behaviour.

In terms of developmental level, the three classes of adaptive functioning correspond to the two main classes of cognitive development. Infants falling behind age-appropriate norms in adaptive behaviour (class 1) also showed decreasing trajectories in cognitive development, with significantly lower scores compared to their peers by age 2. On the other hand, infants with stable or improving adaptive behaviour (classes 2 and 3) did not differ in terms of developmental level, showing rather stable trajectories around age-appropriate norms. Discrepancy between adaptive and cognitive skills has been found before [14, 47], showing lower and more divergent adaptive skills compared to cognitive level. Our findings suggest that the main differences between decreasing and stable/increasing trajectories in adaptive behaviour might be driven by the differences in cognitive level. Less cognitively able individuals appear to fall behind age-appropriate norms in adaptive behaviour, while more cognitively able individuals show stable or increasing trajectories of adaptive behaviour around age-appropriate norms. Cognitive impairment has been shown to have a negative effect on everyday functioning and the development of adaptive behaviour, even beyond the effect of ASD symptoms [19]. However, the profile of good cognitive abilities with poor adaptive skills, which has been reported before in older children with ASD [18, 20], has not emerged from our study. Such profile might emerge later in life, as we have found in a follow-up of part of the current sample in mid-childhood [14]. This highlights the complexity of the development of adaptive behaviour and its relationship with cognitive development and ASD symptomatology, suggesting the necessity to further investigate adaptive behaviour in infancy.

Although findings are consistent, our study differs in three ways from the recent study of Sacrey et al. [17] on latent trajectories of adaptive behaviour in infancy. First, we identified classes across domains of adaptive behaviour instead of ABC score alone, providing more detailed profiles. Second, we used growth mixture modelling [7] instead of latent class growth curve modelling [29], allowing variation within class to capture heterogeneity across individuals. Third, we examined younger ages by including data at 8 months. This earlier observation added particular value to our findings. In fact, children who later met the criteria for ASD might not simply follow a trajectory of progressive impairment in adaptive skills, but some of them might present even stronger skills in the first year of life compared to other subgroups of infants. Our study has strengths in the relatively large sample of infant siblings (*n* = 240) and the analysis of multiple instruments (MSEL, VABS, ADOS, ADI-R, and SCQ), but it also has limitations. First, although trajectories were separated at the group level, there was still a substantial overlap between classes in individual variation. Second, the three-step approach we used to examine the association of class membership with external variables might underestimate such relationship [48]. This is particularly true when classification errors in the assignment of individual class membership are high, and it might explain the discrepancy between VABS, MSEL, and ADOS found in our study. However, high average class posterior probability was a selection criterion for the best model, reducing the impact of classification errors. Third, adaptive behaviour was assessed on the basis of parent-reported measures; however, class comparisons in terms of cognitive level and symptom severity, assessed by observational measures, enhance our confidence in the identified trajectories. Fourth, follow-up studies should investigate trajectories on a higher number of time-points to improve the estimate of the shape of the trajectory curve and test whether they change at later age. Finally, experimenters’ awareness of risk-group status might lead to ascertainment bias due to more intense surveillance for ASD outcome among high-risk siblings compared to low-risk controls.

## Conclusions

High-risk siblings and low-risk controls could be separated into three latent classes representing declining, improving, and stable trajectories of adaptive behaviour between 8 and 36 months. We observed a dissociation between adaptive behaviour and cognitive development, with the improving class in adaptive behaviour showing stable trajectories of cognitive development around average scores. Furthermore, classes significantly differed in ASD symptoms and clinical outcome at 36 months. High levels of adaptive functioning in the first year of life followed by a failure to keep up with age-appropriate norms were linked to higher symptom severity across the social, communication, and repetitive behaviours domains. Furthermore, it was linked to increased likelihood of meeting diagnostic criteria for ASD in toddlerhood. Our findings provide better insight into the variety of paths leading to different functional outcomes within ASD. The identified subgroups indicate homogeneous classes of infants in terms of progression of adaptive functioning over time. These subgroups might be more relevant target groups for intervention aimed at improving later functioning.

## Additional file

Additional file 1: **Table S1.** Descriptive statistics by risk group. **Table S2.** Model fitting. (DOCX 37 kb)

## Abbreviations

ABC: Adaptive Behaviour Composite score (VABS)
ADI-Comm: Communication domain score (ADI-R)
ADI-R: Autism Diagnostic Interview-Revised
ADI-RBI: Repetitive Behaviors and Interests domain score (ADI-R)
ADI-Soc: Social domain score (ADI-R)
ADOS: Autism Diagnostic Observation Schedule
AIC: Akaike information criterion
ASD: Autism spectrum disorder
BIC: Bayesian information criterion
CI: Confidence Interval
Comm: Communication score (VABS)
CSS: Calibrated Severity Score (ADOS)
CSS-RRB: Calibrated Severity Score in the Restricted and Repetitive Behaviors domain (ADOS)
CSS-SA: Calibrated Severity Score in the Social Affect domain (ADOS)
CSS-Tot: Total Calibrated Severity Score (ADOS)
DL: Daily Living score (VABS)
EL: Expressive Language Score (MSEL)
ELC: Early learning composite score (MSEL)
FM: Fine Motor score (MSEL)
GM: Gross Motor score (MSEL)
HR: High-risk siblings
HR-ASD: Siblings with ASD
HR-Atyp: Atypically developing siblings (no ASD)
HR-Typ: Typically developing siblings
LR: Low-risk controls
Mot: Motor score (VABS)
MSEL: Mullen Scales of Early Learning
OR: Odds ratio
RL: Receptive Language score (MSEL)
SCQ: Social Communication Questionnaire
SCQ-Tot: Total score (SCQ)
SD: Standard Deviation
Soc: Socialization score (VABS)
VABS-II: Vineland Adaptive Behavior Scales
VR: Visual Reception score (MSEL)
