# Supplementary material for: Latent trajectories of adaptive behaviour in infants at high and low familial risk for autism spectrum disorder
Source: Mol Autism. 2019 Mar 15;10:13. doi: 10.1186/s13229-019-0264-6 (PMC6420730; doi:10.1186/s13229-019-0264-6)
Supplement: Supplementary file 1 — Table S1. Descriptive statistics by risk group. Table S2. Model fitting. (DOCX 37 kb) [file 13229_2019_264_MOESM1_ESM.docx]

**Supplementary Material**

Latent trajectories of adaptive behaviour in infants at high and low familial risk for autism spectrum disorder

Bussu G., Jones E.J.H., Charman T., Johnson M.H., Buitelaar J.K. and the BASIS Team.

**Contents:**

**Table S1:** Descriptive statistics by risk group.

**Table S2:** Model fitting.

| **Table S1. Descriptive statistics by risk group.** | | | | |
| --- | --- | --- | --- | --- |
|  | **Low-risk controls** | | **High-risk siblings** | |
|  | **mean** | **sd** | **mean** | **sd** |
| **Age** |  |  |  |  |
| **8m** | 7.8 | 1.4 | 8.2 | 1.2 |
| **14m** | 14.3 | 1.3 | 14.5 | 1.3 |
| **24m** | 24.2 | 0.9 | 25.4 | 2.0 |
| **36m** | 38.3 | 2.6 | 39.0 | 3.0 |
| **VABS** |  |  |  |  |
| **Comm 8m** | 101.7 | 13.6 | 93.6 | 16.2 |
| **Comm 14m** | 101.5 | 9.8 | 94.5 | 14.0 |
| **Comm 24m** | 109.4 | 11.8 | 101.1 | 12.7 |
| **Comm 36m** | 108.5 | 10.5 | 97.9 | 14.3 |
| **DL 8m** | 102.5 | 13.6 | 99.0 | 13.6 |
| **DL 14m** | 99.1 | 11.1 | 93.5 | 13.6 |
| **DL 24m** | 109.6 | 11.3 | 103.8 | 13.2 |
| **DL 36m** | 108.6 | 7.9 | 100.8 | 14.0 |
| **Mot 8m** | 96.3 | 13.9 | 86.8 | 16.5 |
| **Mot 14m** | 104.2 | 11.1 | 98.6 | 13.2 |
| **Mot 24m** | 103.6 | 9.0 | 98.5 | 11.3 |
| **Mot 36m** | 99.3 | 10.7 | 91.4 | 12.3 |
| **Soc 8m** | 102.8 | 12.6 | 98.8 | 12.7 |
| **Soc 14m** | 100.6 | 10.7 | 96.4 | 11.9 |
| **Soc 24m** | 106.8 | 10.6 | 98.2 | 11.0 |
| **Soc 36m** | 105.2 | 8.0 | 94.6 | 13.3 |
| **MSEL** |  |  |  |  |
| **ELC 8m** | 107.4 | 12.6 | 102.7 | 15.8 |
| **ELC 14m** | 105.4 | 15.1 | 95.5 | 15.5 |
| **ELC 24m** | 115.6 | 14.2 | 99.8 | 20.2 |
| **ELC 36m** | 117.4 | 15.6 | 103.4 | 24.1 |
| **ADOS at 36m^1^** |  |  |  |  |
| **CSS-Tot** | 2.64 | 2.0 | 3.09 | 2.56 |
| **CSS-SA** | 3.31 | 2.26 | 3.45 | 2.62 |
| **CSS-RRB** | 3.67 | 2.46 | 4.57 | 2.63 |
| **ADI-R at 36m^2^** |  |  |  |  |
| **ADI-Comm** | 1.44 | 1.49 | 4.04 | 4.69 |
| **ADI-Soc** | 1.48 | 1.42 | 3.78 | 4.93 |
| **ADI-RBI** | 0.41 | 0.64 | 1.48 | 2.26 |
| **SCQ at 36m^2^** |  |  |  |  |
| **SCQ-Tot** | 2.88 | 2.35 | 6.40 | 6.90 |
| **Gender** |  | |  | |
| **Female** | 40 (54%) | | 82 (49%) | |
| **Male** | 34 (46%) | | 84 (51%) | |
| *Note.*  This table shows descriptive statistics by risk group for the entire sample.  *Abbreviations.* VABS = Vineland Adaptive Behavior Scales; Comm = communication score; DL = daily living score; Mot = motor score; Soc = socialization score; MSEL = Mullen Scales of Early Learning; ELC = early learning composite score; ADOS = Autism Diagnostic Observation Schedule; CSS = calibrated severity score; ADI-R = Autism Diagnostic Interview-Revised; ADI-Comm = Communication domain score (ADI-R); ADI-Soc = Social domain score (ADI-R); ADI-RBI = Restricted Behaviors and Interests domain score (ADI-R); SCQ = Social Communication Questionnaire; SCQ-Tot = Total score (SCQ);  ^1^ Data were available for a subsample of n=235 infants.  ^2^ Data were available for a subsample of n=239 infants. | | | | |

| **Table S2: Model fitting.** | | | | | | | | | | | |
| --- | --- | --- | --- | --- | --- | --- | --- | --- | --- | --- | --- |
|  | c | BIC | AIC | loglik | Pr | n1 | n2 | n3 | n4 | n5 | n6 |
| Common/diagonal variance matrix | |  |  |  |  |  |  |  |  |  |  |
|  | 1l | 28665.76 | 28610.07 | -14289.0 | - | 240 | - | - | - | - | - |
|  | 1q | 28593.31 | 28530.66 | -14247.3 | - | 240 | - | - | - | - | - |
|  | 2l | 28610.63 | 28534.06 | -14245.0 | 0.87 | 73 | 167 | - | - | - | - |
|  | 2q | 28528.49 | 28438 | -14193.0 | 0.85 | 100 | 140 | - | - | - | - |
|  | 3l | 28608.76 | 28511.3 | -14227.6 | 0.91 | 99 | 6 | 135 | - | - | - |
|  | 3q | 28522.89 | 28404.55 | -14168.3 | 0.90 | 88 | 149 | 3 | - | - | - |
|  | 4l | 28620.78 | 28502.44 | -14217.2 | 0.88 | 7 | 108 | 118 | 7 | - | - |
|  | 4q | 28522.98 | 28376.79 | -14146.4 | 0.92 | 8 | 172 | 3 | 57 | - | - |
|  | 5l | 28634.85 | 28495.62 | -14207.8 | 0.86 | 17 | 8 | 104 | 105 | 6 | - |
|  | 5q | 28546.99 | 28372.96 | -14136.5 | 0.86 | 8 | 27 | 3 | 137 | 65 | - |
|  | 6l | 28646.49 | 28486.38 | -14197.2 | 0.90 | 141 | 3 | 10 | 48 | 6 | 32 |
|  | 6q | 28582.72 | 28380.84 | -14132.4 | 0.89 | 1 | 83 | 38 | 3 | 107 | 8 |
| Common/not diagonal variance matrix | |  |  |  |  |  |  |  |  |  |  |
|  | 1l | 28583.86 | 28517.73 | -14239.9 | - | 240 | - | - | - | - | - |
|  | 1q | 28474.11 | 28390.58 | -14171.3 | - | 240 | - | - | - | - | - |
|  | 2l | 28571.96 | 28484.94 | -14217.5 | 0.95 | 15 | 225 | - | - | - | - |
|  | 2q | 28470.90 | 28359.52 | -14147.8 | 0.95 | 227 | 13 | - | - | - | - |
|  | 3l | 28587.99 | 28480.09 | -14209.0 | 0.88 | 39 | 189 | 12 | - | - | - |
|  | 3q | 28485.11 | 28345.88 | -14132.9 | 0.97 | 13 | 224 | 3 | - | - | - |
|  | 4l | 28608.42 | 28479.64 | -14202.8 | 0.90 | 12 | 185 | 3 | 40 | - | - |
|  | 4q | 28505.28 | 28338.21 | -14121.1 | 0.93 | 189 | 37 | 3 | 11 | - | - |
|  | 5l | 28631.39 | 28481.72 | -14197.9 | 0.90 | 74 | 3 | 32 | 119 | 12 | - |
|  | 5q | 28544.80 | 28349.88 | -14118.9 | 0.93 | 185 | 1 | 15 | 36 | 3 | - |
|  | 6l | 28649.22 | 28478.67 | -14190.3 | 0.90 | 42 | 64 | 6 | 111 | 5 | 12 |
|  | 6q | 28569.67 | 28346.91 | -14109.5 | 0.87 | 66 | 4 | 13 | 11 | 31 | 115 |
| Class-specific/diagonal variance matrix | |  |  |  |  |  |  |  |  |  |  |
|  | 1l | 28665.76 | 28610.07 | -14289.0 | - | 240 | - | - | - | - | - |
|  | 1q | 28593.31 | 28530.66 | -14247.3 | - | 240 | - | - | - | - | - |
|  | 2l | 28601.35 | 28521.3 | -14237.6 | 0.88 | 163 | 77 | - | - | - | - |
|  | 2q | 28522.85 | 28428.87 | -14187.4 | 0.87 | 135 | 105 | - | - | - | - |
|  | 3l | 28605.06 | 28500.64 | -14220.3 | 0.89 | 157 | 10 | 73 | - | - | - |
|  | 3q | 28523.12 | 28397.82 | -14162.9 | 0.87 | 20 | 177 | 43 | - | - | - |
|  | 4l | 28612.39 | 28483.61 | -14204.8 | 0.90 | 135 | 11 | 88 | 6 | - | - |
|  | 4q | 28524.58 | 28367.95 | -14139.0 | 0.85 | 134 | 37 | 4 | 65 | - | - |
|  | 5l | 28638.78 | 28485.63 | -14198.8 | 0.84 | 88 | 105 | 30 | 6 | 11 | - |
|  | 5q | 28556.29 | 28368.34 | -14130.2 | 0.83 | 117 | 6 | 30 | 79 | 8 | - |
|  | 6l | 28666.19 | 28488.68 | -14193.3 | 0.86 | 45 | 129 | 42 | 11 | 6 | 7 |
|  | 6q | 28591.30 | 28372.01 | -14123.0 | 0.85 | 123 | 34 | 19 | 4 | 59 | 1 |
| Class-specific/not diagonal variance matrix | |  |  |  |  |  |  |  |  |  |  |
|  | 1l | 28583.86 | 28517.73 | -14239.9 | - | 240 | - | - | - | - | - |
|  | 1q | 28474.11 | 28390.58 | -14171.3 | - | 240 | - | - | - | - | - |
|  | 2l | 28578.27 | 28487.77 | -14217.9 | 0.83 | 177 | 63 | - | - | - | - |
|  | 2q | 28475.58 | 28360.72 | -14147.4 | 0.85 | 188 | 52 | - | - | - | - |
|  | 3l | 28599.08 | 28484.22 | -14209.1 | 0.85 | 119 | 85 | 36 | - | - | - |
|  | 3q | 28484.42 | 28338.23 | -14127.1 | 0.90 | 171 | 65 | 4 | - | - | - |
|  | 4l | 28616.55 | 28477.32 | -14198.7 | 0.90 | 41 | 12 | 182 | 5 | - | - |
|  | 4q | 28521.22 | 28343.71 | -14120.9 | 0.93 | 68 | 2 | 168 | 2 | - | - |
|  | 5l | 28646.30 | 28482.71 | -14194.4 | 0.81 | 159 | 38 | 27 | 4 | 12 | - |
|  | 5q | 28557.60 | 28348.76 | -14114.4 | 0.89 | 169 | 24 | 5 | 41 | 1 | - |
|  | 6l | 28681.00 | 28493.05 | -14192.5 | 0.89 | 36 | 161 | 23 | 2 | 6 | 12 |
|  | 6q | 28602.14 | 28361.98 | -14112.0 | 0.85 | 5 | 24 | 115 | 44 | 41 | 11 |
| *Note.*  This table shows the metrics of model fitting for the different models tested based on the polynomial degree of the growth curve, the variance/covariance matrix and the number of classes. Separate sections illustrate results for models differing in variance/covariance matrices across classes (common vs class-specific, and diagonal vs non-diagonal matrices). Each section shows results for linear and quadratic growth with 1 to 6 classes. The 3-class quadratic model with class-specific and diagonal variance/covariance matrix for random effects was the one selected as best model for further analysis.  *Abbreviations.* c = number of classes; BIC = Bayesian Information Criterion; AIC = Akaike Information Criterion; loglik = log-likelihood; Pr = average class posterior probability; n1-n6 = number of infants in each class. | | | | | | | | | | | |
